# Quantification of retinal blood leakage in fundus fluorescein angiography in a retinal angiogenesis model

**DOI:** 10.1038/s41598-021-99434-2

**Published:** 2021-10-06

**Authors:** Cesar H. Comin, Demetrios I. Tsirukis, Ye Sun, Xiaoyin Xu

**Affiliations:** 1 Department of Computer Science, Federal University of São Carlos, São Carlos, SP Brazil; 2 Department of Ophthalmology, Boston Children’s Hospital, Harvard Medical School, 300 Longwood Avenue, Boston, MA 02115 USA; 3 Department of Radiology, Brigham and Women’s Hospital, Harvard Medical School, Boston, MA USA

**Keywords:** Computational biology and bioinformatics, Neuroscience

## Abstract

Blood leakage from the vessels in the eye is the hallmark of many vascular eye diseases. One of the preclinical mouse models of retinal blood leakage, the very-low-density-lipoprotein receptor deficient mouse (*Vldlr*^*−/−*^), is used for drug screening and mechanistic studies. Vessel leakage is usually examined using Fundus fluorescein angiography (FFA). However, interpreting FFA images of the *Vldlr*^*−/−*^ model is challenging as no automated and objective techniques exist for this model. A pipeline has been developed for quantifying leakage intensity and area including three tasks: (i) blood leakage identification, (ii) blood vessel segmentation, and (iii) image registration. Morphological operations followed by log-Gabor quadrature filters were used to identify leakage regions. In addition, a novel optic disk detection algorithm based on graph analysis was developed for registering the images at different timepoints. Blood leakage intensity and area measured by the methodology were compared to ground truth quantifications produced by two annotators. The relative difference between the quantifications from the method and those obtained from ground truth images was around 10% ± 6% for leakage intensity and 17% ± 8% for leakage region. The Pearson correlation coefficient between the method results and the ground truth was around 0.98 for leakage intensity and 0.94 for leakage region. Therefore, we presented a computational method for quantifying retinal vascular leakage and vessels using FFA in a preclinical angiogenesis model, the *Vldlr*^*−/−*^ model.

## Introduction

In many vascular eye diseases, the hallmarks of pathological abnormalities are morphological changes in the form of unhealthy blood vessels and blood leakage from the vessels. In conditions like retinopathy of prematurity, age-related macular degeneration (AMD), and diabetic retinopathy, abnormal blood vessels grow and may leak blood in the retina to eventually cause retinal detachment and blindness^1–3^. Although current anti-VEGF (vascular endothelial growth factor) therapies have received remarkable success in treating vascular eye diseases^4,5^, some patients are non-responsive. To develop new treatments, preclinical animal models have become crucial for drug screening as the models can mimic the unhealthy blood vessels and vessel leakage seen in human vascular eye diseases. Blood leakage in preclinical animal models is used as a parameter to evaluate new antiangiogenic drugs for these vascular eye diseases.

The very-low-density-lipoprotein receptor deficient mouse (*Vldlr*^*−/−*^) is one of preclinical mouse models of pathological angiogenesis with retinal vascular leakage—features similar to human neovascular AMD^6–8^. In this mouse model, abnormal vessels grow into photoreceptor avascular areas and immune privilege in subretinal space is broken down^8^. Therefore, this is a suitable model to study neuroinflammation-vascular crosstalk occurring in the AMD. Low fatty acid uptake and high circulating lipid level in the retina of *Vldlr*^*−/−*^ make this model a perfect model for metabolism-dependent vascular dysfunction occurring in the AMD^9^. In addition, because of high level of VEGF in the retina of *Vldlr*^*−/−*^ mice, this mouse model can be used for screening of inhibitors of VEGF. Therefore, there is a pressing need for a method to quantify vascular changes, especially blood leakage in this mouse model. Fundus fluorescein angiography (FFA) is a powerful in vivo imaging technique that is widely used for studying retinal blood leakage in the retina^10,11^. In FFA, a fluorescein dye is injected into the animal and a special camera records the dye-labeled blood leakage. With FFA, the blood leakage can be examined by imaging at different time points. Leakage regions can then be manually marked and used for quantitative assessment^12^. Nevertheless, manual quantification methods may introduce human bias and tend to be time-consuming; therefore, a robust computer-based quantification method would greatly benefit FFA analysis. In this work, we present an algorithm for segmenting and quantifying blood leakage regions around blood vessels in FFA imaging of a preclinical model, *Vldlr*^*−/−*^ mice.

Some methods have been reported for other retinal blood leakage mouse or rat models. For example, Wigg et al. used off-the-shelf software with FFA images from a mouse model to manually quantify blood leakage from choroidal neovascularization (CNV) by delineating the boundaries of CNV and chorioretinal burns lesion areas in FFA images from a mouse model^13^. Criswell et al. manually quantified blood leakage in FFA images by measuring the diameter of choroidal neovascular membrane as being a representation of their sizes^14^. The limitations of the manual approach include observer bias, inter-observer variations, and the long time required for labelling the samples. To obtain unbiased quantification of FFA images, Guthrie et al. developed a multi-Otsu thresholding algorithm for segmenting blood leakage from CNV^15^. Hui et al. presented a combination of automated and manual approaches to analyze blood leakage in video-based FFA by applying principal component analysis to register frames of the video for pixel-based intensity analysis^16^. Their methodology was able to enhance leakage regions, but it was not aimed at detection or segmentation of the regions. Also, blood vessels were manually segmented. The authors used the same methodology in a recent study regarding the comparison of fluorescein angiography dynamics between retinal and cortical vessels^17^. In another study aimed at quantifying blood leakage from laser-induced CNV, Toma et al. compared the segmentation given by Adobe Photoshop and Image-Pro Plus and found little difference in the results, reflecting a degree of equivalency in the methods^18^. To perform the analysis, they needed to carefully choose parameters to achieve satisfactory results, which may introduce user bias and may not generalize well to other data. Zhao et al. observed that leakage regions in human FFA tend to be salient with respect to the rest of the image, that is, they tend to be different from their surrounding context^19^. Thus, they used saliency maps for identifying leakage regions and focused on counting leakage regions on Malarial Retinopathy samples.

In this work, we present a robust image-processing pipeline for detecting and segmenting blood leakage and separating the leakage from blood vessels in time-varying animal FFA images of *Vldlr*^*−/−*^ mouse model. The focus of our method is to allow precise quantification of the intensity and area of blood leakage regions, so that the time evolution of the leakage can be evaluated. Thus, our approach focuses on removing blood vessels from the image while avoiding changes to the appearance of leakage regions, so that they can be correctly analyzed. The method combines morphological and log-Gabor quadrature filters for identifying leakage regions, which can then be used in conjunction with blood vessels segmentation using quadrature filters and the Chan–Vese method^20^ for characterizing fluorescein fundus images. In addition, a reference point identification approach based on large vessels detected in the images is defined and used for image registration. The results were then used for calculating the time evolution of leakage regions.

## Materials and methods

### Animals

All animal studies were conducted in accordance with the Association for Research in Vision and Ophthalmology Statement for the Use of Animals in Ophthalmic and Vision Research and performed according to protocols approved by the Institutional Animal Care and Use Committee (IACUC) at the Boston Children’s Hospital and the Animal Research Reporting of In Vivo Experiments (ARRIVE) guidelines (PLoS Bio 8(6), e1000412, 2010). Mice were housed under specific pathogen–free conditions. Very low-density lipoprotein receptor heterozygous mice (*Vldlr*^+*/−*^) were purchased from Jackson Laboratory (Stock# 002529) and were bred to generate homozygous (*Vldlr*^*−/−*^).

### FFA imaging

All the FFA images were taken using a retinal-imaging microscope (Micron IV, Phoenix Research Laboratories) from *Vldlr* knockout mice as we described^8^. Mice were anesthetized with a mixture of xylazine and ketamine, and pupils were dilated with topical drops of Cyclomydril (Alcon Laboratories, Fort Worth, TX). Mice were intraperitoneally injected with fluorescein AK-FLU OR (Akorn, Lake Forest, IL) and FFA images were taken at different time points after fluorescein injection.

### Image processing and data analysis

#### Separating blood leakage from blood vessels

**Figure 1:**
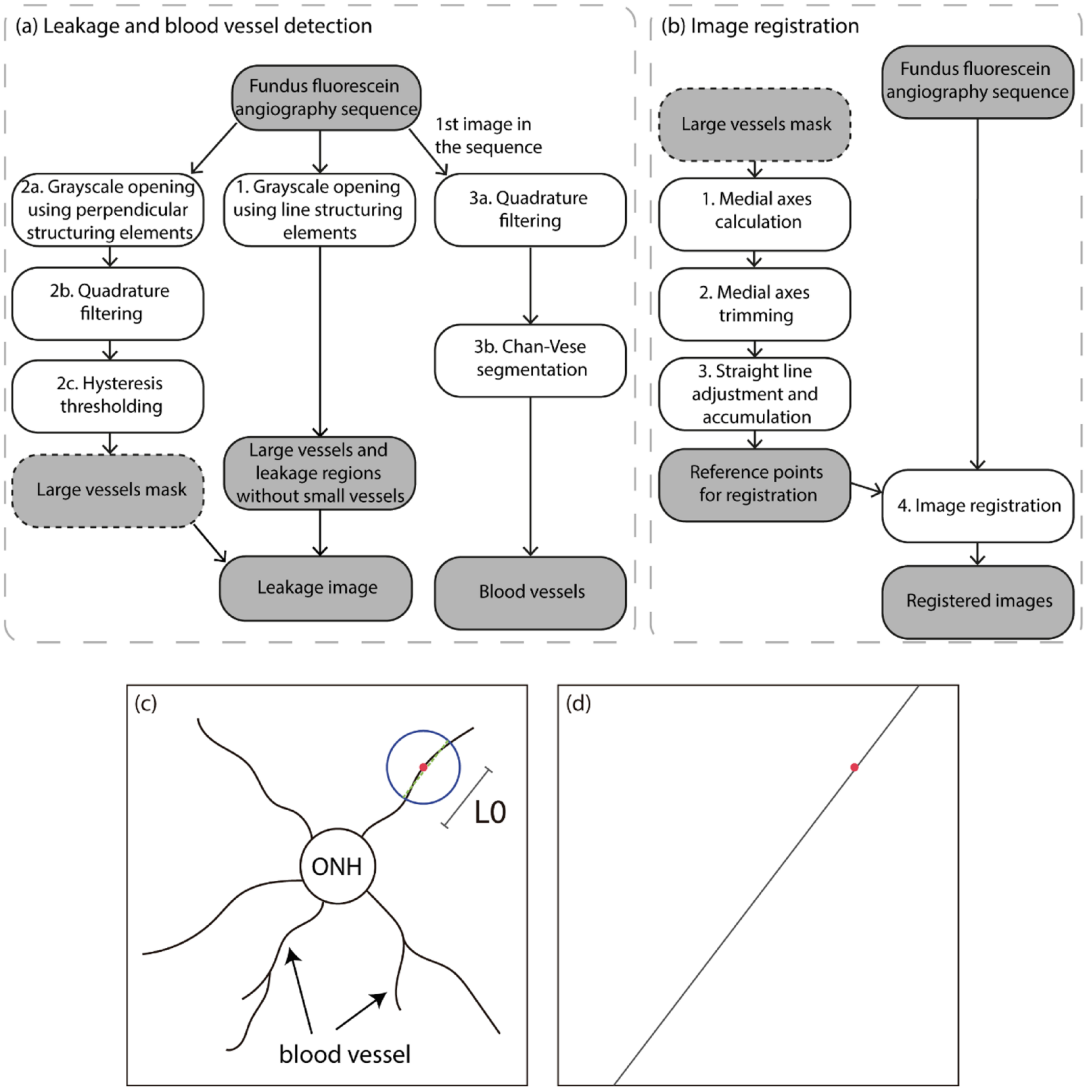
(**a**,**b**) Flowcharts showing the steps of the proposed methodology for (**a**) detecting leakage regions and blood vessels and (**b**) registering the FFA image sequences. White rectangles indicate processing steps and shaded rectangles indicate the respective resulting images. Note that the large vessels mask image is used as input for image registration. (**c**,**d**) Illustration of the methodology used for detecting a stable point inside the optic disk. (**c**) Linear regression applied to the neighborhood of a pixel (red point) in the medial axis of a blood vessel. The pixels used in the calculation are those inside the top circle. The dashed line in top circle indicates the obtained line. (**d**) The line is drawn in the accumulator array. The dot indicates the original point for reference. ONH: optic nerve head; ${L}_{0}$ defines a scale of analysis for applying the linear regression.

To characterize blood leakage regions, we first remove blood vessels from the image with the assumption that, by removing tubular structures, the remaining structures will be related to leakage regions. Since the image contains blood vessels having widely varying scales, the blood vessel removal was done separately for small vessels and large vessels. A diagram containing the steps of the methodology is shown in Fig. 1a. Small vessels were removed by applying grayscale-opening operations using line structuring elements at different angles. Defining as $I_{{\theta_{i} }}$ the resulting image from the grayscale opening applied at angle $\theta_{i}$, the final image was calculated as

1 $$ \widetilde{{I_{l} }}\left( {x,y} \right) = \mathop {\min }\limits_{{\theta_{i} }} \left\{ {I_{{\theta_{i} }} \left( {x,y} \right)} \right\} $$

where $\left( {x,y} \right)$ represent the coordinates of a pixel and $\theta_{i}$ is uniformly distributed over 180 degrees such that $\theta_{j} = \left( {j - 1} \right) \times \frac{\pi }{8}, j = 1, \ldots ,8$ . The motivation for this approach was that the final values in image $\widetilde{{I_{l} }}$ will be calculated as the minimum value along a perpendicular line to the blood vessel, centered at the blood vessel. The size of the structuring element, $I_{sv}$, defined the diameter of the blood vessels to be removed. Its length should be slightly larger than the diameter of the thickest vessel to be removed in this step.

The procedure for removing large vessels tries to avoid the removal of leakage regions. First, a methodology was applied for enhancing large blood vessels. We tested various blood vessel enhancement methods such as the Frangi filter^21^ and wavelets^22^. Good results were obtained for a combination of morphological operations and quadrature filters^23^. For the morphological operations, a set of line structuring elements $S_{{\theta_{i} }}$ having angles in the range $\left[ {0,\pi } \right]$ is defined. A respective perpendicular line structuring element $S_{{\theta_{i} }}^{ \bot }$ is defined for each $S_{{\theta_{i} }}$. A grayscale opening operation was applied using each structuring element. Defining $\tilde{I}_{{\theta_{i} }}$ and $\tilde{I}_{{\theta_{i} }}^{ \bot }$ as the result of the grayscale opening for the structuring elements $S_{{\theta_{i} }}$ and $S_{{\theta_{i} }}^{ \bot }$, respectively, a difference image is calculated as $\Delta I_{{\theta_{i} }} = \tilde{I}_{{\theta_{i} }} - \tilde{I}_{{\theta_{i} }}^{ \bot }$. This difference image should have large values if $S_{{\theta_{i} }}$ is aligned with the blood vessel. Otherwise, at leakage regions, both morphological operations will result in similar values, and the difference image will have low values. Therefore, leakage regions are suppressed.

A final image combining the analysis at all angles was calculated as

2 $$ \tilde{I}_{v} \left( {x,y} \right) = \mathop {\max }\limits_{{\theta_{i} }} \left\{ {\Delta I_{{\theta_{i} }} \left( {x,y} \right)} \right\}. $$

This image had leakage regions suppressed, but blood vessels might still have non-uniform intensities. Therefore, a quadrature filter was applied on the result. We considered a set of log-Gabor filters and followed the same procedure as in^23^, which is described in the next section. The final image was represented as $I_{v}$.

To combine images $\tilde{I}_{l}$ and $I_{v}$, a hysteresis threshold was applied to image $I_{v}$. Large blood vessels tend to be clearly visible on fluorescein images. Therefore, the accuracy of the segmentation was not particularly sensitive to the values used for the hysteresis threshold. Blood vessels segmented in image $I_{v}$ were then removed from image $\tilde{I}_{l}$, defining the final leakage image $I_{l}$.

#### Blood vessel segmentation

In the previous section, removing blood vessels without altering the appearance of leakage regions allowed the characterization of leakage intensity and areas. We defined a specific procedure to remove as many blood vessels as possible while following the above constraint. In this section, we focus on segmenting the blood vessels.

The blood vessel segmentation followed the methodology described in^24^. First, we applied a set of log-Gabor quadrature filters that were generated so as to have optimal coverage of the Fourier domain using the techniques presented in^25^. For each scale index $s \in \left\{ {1,2, \ldots ,n_{s} } \right\}$ the center frequency of the respective filters is given by $w_{0} = 2^{{\rho_{s} }} /N$, where $N$ is the size of the image that is typically two to the power of an integer, such as 256 and 512, and $\rho_{s}$ is calculated as

3 $$ \rho_{s} = log_{2} \left( N \right) - s $$

with $s$ being the index of the scale to allow the generation of a multiscale log-Gabor filter bank. A bandwidth of 2 octaves is used for the filter bank^24^. The remaining calculations follow the computation of the vesselness map defined in^23^. We designed eight filters that are oriented uniformly over 180 degrees, such that the orientation of the $j$-th filter is $\theta_{j} = \left( {j - 1} \right) \times \frac{\pi }{8}, j = 1, \ldots ,8.$ The combined response at all orientations is given by

4 $$ q_{n} = \mathop \sum \limits_{j = 1}^{8} q_{n}^{j} \;{\text{for}}\;n = 1, \ldots ,n_{s} $$

where $q_{n}^{j}$ is the result of applying a quadrature filter at scale $n$ and orientation $\theta_{j}$. The responses at the different scales are combined to define an overall response to the filter bank^24^, given as

5 $$ P = \frac{{\mathop \sum \nolimits_{n = 1}^{{n_{s} }} q_{n} |q_{n} |^{3} }}{{\mathop \sum \nolimits_{n = 1}^{{n_{s} }} |q_{n} |^{3} }}. $$

The final vesselness map is then given as the real part of a regularized version of $P$^24^

6 $$ LP = {\text{real}}\left\{ {\frac{P\left| P \right|}{{|P|^{2} + \sigma^{2} }}} \right\} $$

where $\sigma$ reduces artifacts caused by noise. In the experiments we found that, provided that $\sigma > 1$, this parameter does not significantly impact on the results. Therefore, we set $\sigma = 3$ for all experiments. Following the quadrature filters application, a Chan-Vese graph cut segmentation^20^ is applied to the enhanced blood vessels image.

#### Reference point detection

To compare the images taken at different time points, we defined a reference point that can be precisely located for all images to help the registration of the images (described in the next section) for characterizing leakage regions and blood vessel properties. Based on calculating the medial axes of the blood vessels and defining a respective graph describing the topology of the vessels, we defined a method to locate the convergence points of the blood vessels that are close to the optic disk. Then, a procedure for simplifying the obtained graph and prolongations of the blood vessel segments was used to locate the convergence point of the vessels. A diagram illustrating the steps of the methodology is shown in Fig. 1b.

We started with the binary image obtained after applying the hysteresis threshold to image $I_{v}$, as described in Sect. “Separating blood leakage from blood vessels”. The medial lines of the blood vessels were obtained using a thinning procedure^26^. The obtained structures, the skeletons of the blood vessels, were then represented using a graph. Each bifurcation and termination of the skeleton was represented as a node, and the nodes were connected if between them was a blood vessel segment. Bifurcations and terminations were defined as skeleton pixels having, respectively, at least three and exactly one neighbor. The positions of the pixels of the medial axis associated to each edge were also stored in the graph structure. Small skeleton branches were then iteratively removed, meaning branches having lengths shorter than a threshold value $T_{b}$ are removed. This procedure might generate new small branches, which were also removed iteratively until there were no more branches smaller than $T_{b}$.

Each edge of the final graph was associated with a medial axis segment and many of these segments were oriented towards the optic disk. Therefore, a straight line that has been adjusted to a segment is likely to pass over the optic disk. The crossing point between a number of such straight lines should represent a stable reference point for a set of time-varying image samples. This point was detected as follows. For each point $$p_{i}$$ of a segment, a linear regression was applied to all points belonging to the same segment that are also inside a circle of radius $$L_{0}$$ centered on point $$p_{i}$$, as illustrated in Fig. 1c. $$L_{0}$$ defined a scale of analysis for applying the linear regression. The result was a respective line $$l_{i} $$ that can be used for describing the local neighborhood of point $$p_{i}$$. An empty accumulator array $$A$$ having the same size as the original image was created, and line $$l_{i}$$ was drawn on this accumulator array, as illustrated in Fig. 1d. The process was repeated for all points of all segments. The position of the maximum value of accumulator $$A$$ represented the main convergence point of the blood vessels.

#### Image registration

Since the images are taken at different time points, the field of view, also called Region of Interest (ROI), might change among images and was corrected by registering the images into a fixed coordinate frame. Since the images at early time points always contained the smallest leakage region and less fluorescein saturation, all images were registered to the first time point. The main source of variation between the images is caused by translation and rotation. Therefore, we registered the images using a rigid transformation. A cross-correlation similarity metric was used for comparing the pair of the images to be registered, and the parameters of the rigid transformation are optimized using a gradient descent.

The whole process was made more robust and efficient by using the reference points identified using the methodology described in the previous section. That is, the image to be registered is first translated so that its reference point coincides with the same point found for the reference image.

## Results

### Blood leakage identification and blood vessel segmentation

**Figure 2:**
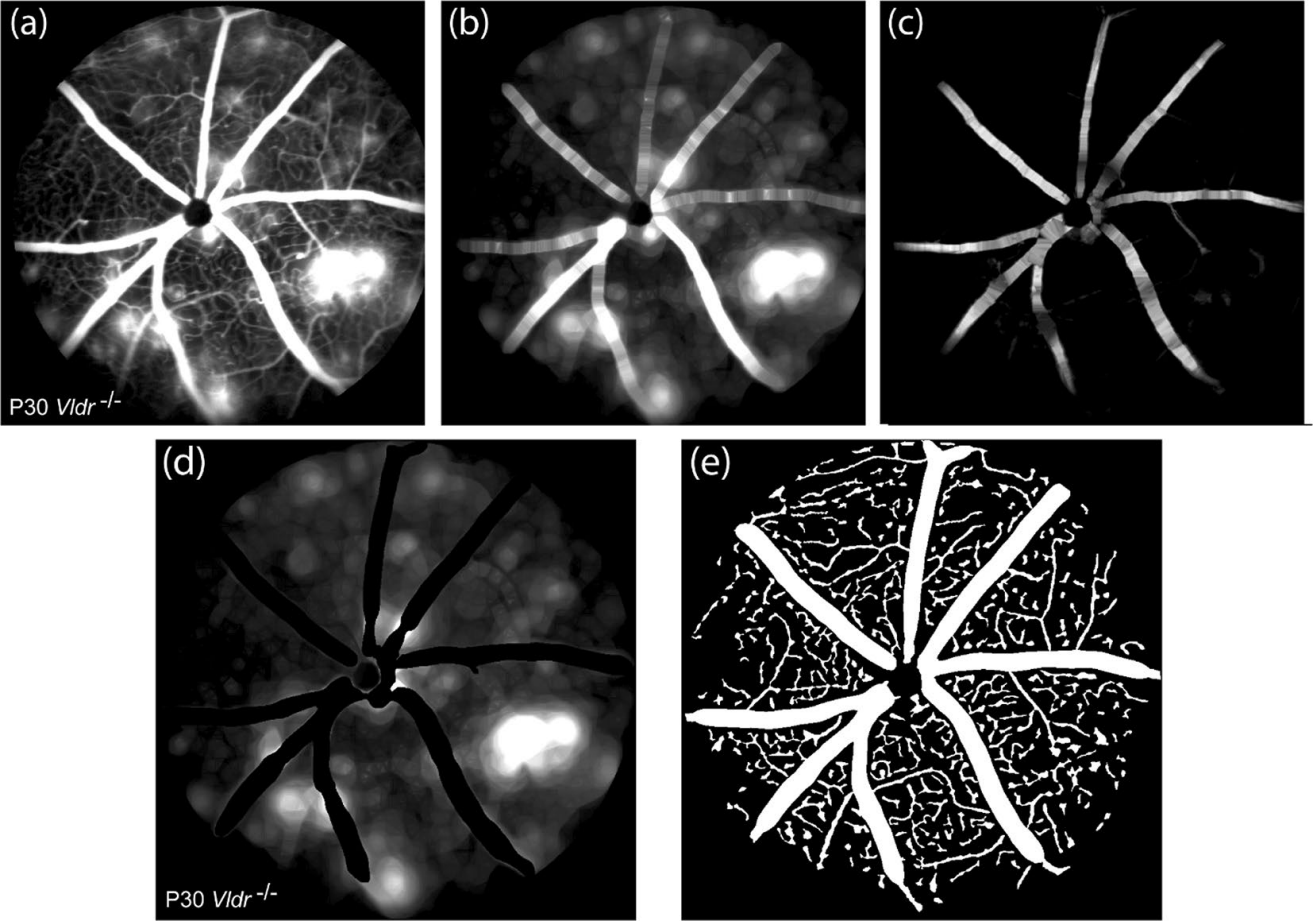
(**a**)–(**c**) Representative results of the main steps of the blood leakage quantification methodology. (**a**) Original image, (**b**) image ${{\tilde{I }}_{l}}$ obtained after small vessel removal, (**c**) image  ${\tilde{I }}_{v}$ obtained after grayscale opening using orthogonal structuring elements. (**d**,**e**) Representative images obtained from our method. (**d**) Blood leakage image obtained after small and large vessel removal. (**e**) Segmented blood vessels.

The first step of the methodology is removing small and large blood vessels from the image. Figure 2 shows a typical result from this step. Grayscale opening operations using line-structuring elements were applied to the original image, Fig. 2a, resulting in an image $\tilde{I}_{l}$ having small blood vessels removed, as shown in Fig. 2b. Leakage regions became slightly blurred, but their intensity was still very similar to those contained in the original image. Next, large blood vessels were identified by applying grayscale opening operations using perpendicular line-structuring elements to the original image and calculating the respective residue ${\Delta }I_{{\theta_{i} }}$ for each angle $\theta_{i}$. As indicated by Eq. (2), the maximum of ${\Delta }I_{{\theta_{i} }}$, represented as $\tilde{I}_{v}$, among all angles should be high for large blood vessels and low for small vessels, leakage regions and the background. Figure 2c shows the result of this step for the original image shown in Fig. 2a. Images $\tilde{I}_{l}$ and $\tilde{I}_{v}$ were combined to define the final leakage image $I_{l}$, which is shown in Fig. 2d.

The next step of the methodology is applying the procedure for segmenting blood vessels. For the quadrature filter, two scales were necessary for defining the response $P$ indicated in Eq. (4). The obtained segmentation of Fig. 2a is shown in Fig. 2e. Large blood vessels and the majority of small vessels were correctly segmented. The identified leakage regions and blood vessels are quantified at the next step.

### Image registration and blood leakage quantification

**Figure 3:**
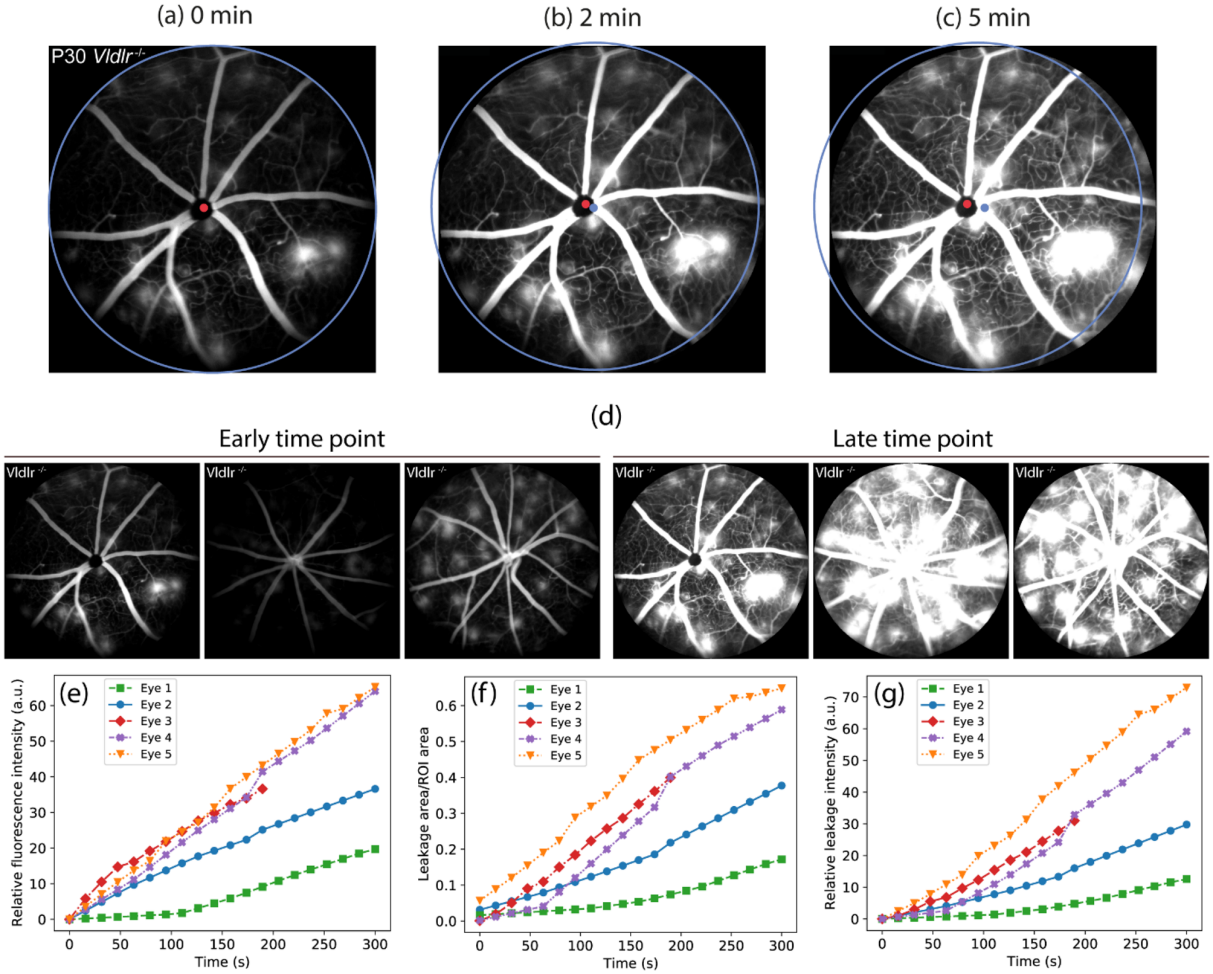
(**a**)–(**c**)Reference point, shown in red, obtained for FFA images from *Vldlr*^*−/−*^ mice at different time points. The blue dot indicates the pixel position of the red dot in (**a**) for reference. The blue circle indicates the position of the ROI of the first time point on the subsequent time points and can be used as a visual guide for the movement of the sample along time. (**d**–**f**) Example of FFA leakage quantification allowed by the leakage segmentation methodology. (**d**) Representative FFA images from *Vldlr*^*−/−*^ mice at early and late time points post fluorescein injection, respectively. (**e**) Relative fluorescein intensity as a function of time for FFA images. (**f**) Leakage region divided by ROI area as a function of time calculated for the FFA images (n = 5). (**g**) Relative fluorescein intensity inside the identified leakage regions as a function of time for FFA images (n = 5).

An initial approximation for the registration process was made using the reference optic disk point found using the methodology presented in Sect. “Reference Point Detection”. The final registration was done using the Python library SimpleITK^27^ using the criteria presented in Sect. “Image Registration”. Figure 3a–c show the reference point detected in FFA images at 3 different time points in red. The detected points were located at similar regions in all images, which was near the center of the optic disk. The raw position, in pixel coordinates, of the reference point detected in Fig. 3a is shown as a blue dot in Fig. 3b, c. The blue and red dots do not match in Fig. 3b, c due to the movement of the sample. Using the reference point as a first approximation of the sample movement, the images can then be further registered using the cross-correlation similarity. After registering the images, the movement of the sample can be visualized by overlaying the identified position of the ROI of the first image onto the subsequent images. This is shown as a blue circle in Fig. 3a–c. It is clear that the sample had significant movement along the time points. Therefore, the registration step is fundamental for the correct quantification of blood leakage in the samples.

We then proceed to quantify the intensity and area of blood leakage in the segmented images. Since the correspondence between absolute intensity and fluorescein concentration is usually not known, we considered relative values of detected fluorescein. We calculated the average intensity of all pixels in the blood leakage images subtracted by the same quantity obtained for the first image of the sequence. This means that the first image will always have a relative fluorescein intensity of 0. In addition, to provide values at equally spaced time points for all image sequences, which could be useful for calculating derivatives, the obtained values were linearly interpolated.

Figure 3d–g show the analyzed result for FFA images from different *Vldlr*^*−/−*^ mouse eyes under different time points. The representative FFA image of *Vldlr*^*−/−*^ mice at early and late time points are shown in Fig. 3d. The relative fluorescein intensity of each time sequence is shown in Fig. 3e. The total fluorescein intensity increased linearly with time for these samples. To delineate leakage regions, a threshold $T$ was applied to the images to associate each pixel with either a leakage region or the image background. Here we applied the Otsu’s automatic thresholding method^28^ to delineate leakage regions. It showed that the obtained areas agree with manual segmentations of the samples. The calculated areas were normalized by the area of the ROI. Figure 3f shows normalized leakage regions calculated for FFA images. Leakage rapidly increases for eyes #3, #4 and #5 while eyes #1 and #2 show a slower initial growth. These results provide an unambiguous quantification of the leakage region contained in each FFA sequence.

Another approach for quantifying leakage intensity is to consider only pixels inside the identified leakage regions. This can be done by summing pixel intensities in image $I_{l}$, only after considering pixels inside the regions obtained after the thresholding operation described above. The obtained value was then divided by the ROI area. The result of this quantification is shown in Fig. 3g. The obtained values are similar to those shown in Fig. 3e, with only eye 5 being slightly distinct because the intensity of the pixels in image $I_{l}$ outside the identified leakage regions were low and did not contribute much to the total fluorescence. Therefore, using the average intensity of image $I_{l}$, as done for the results in Fig. 3f, seems to be a better approach since it does not need a thresholding operation.

**Figure 4:**
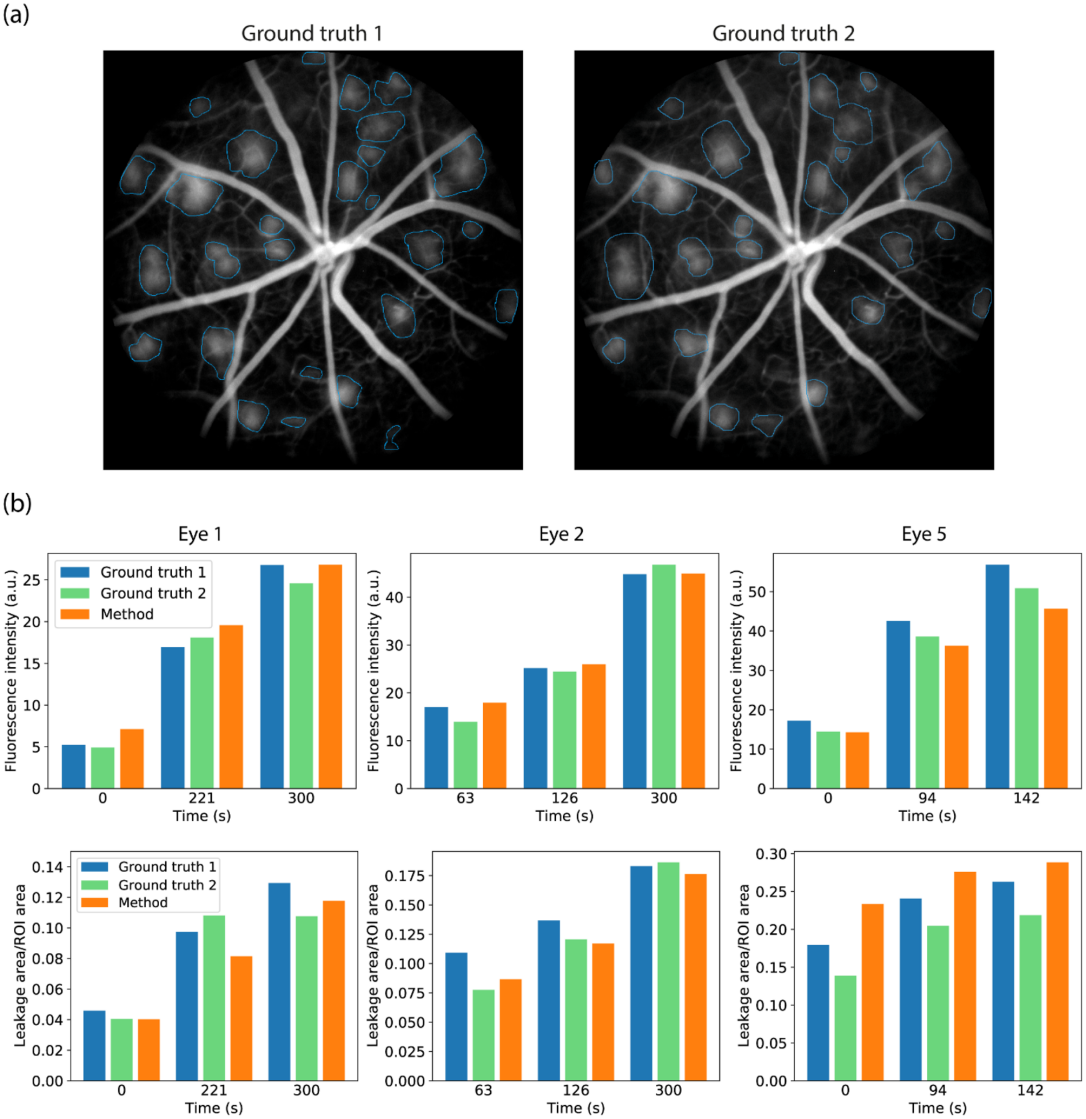
(**a**) Example of manually marked leakage regions from two annotators. These annotations were used as ground truth for assessing the performance of the leakage identification methodology. (**b**) Comparison of leakage intensity (upper row of plots) and area (lower row of plots) between ground truth images produced by two annotators and the results of the method for three time points of three different eyes.

To verify that the method correctly measures leakage intensity and area, two specialists manually annotated leakage regions from 9 images to produce ground truth leakage images. Three images from three different eyes were annotated. Figure 4a shows the annotations of the two specialists for one of the images. The fluorescence intensity and area of the ground truth images were compared to those obtained by the method. In the case of fluorescence intensity, the values were not normalized by the first sample to allow a fair comparison between the results because otherwise the first sample would have zero fluorescence intensity for all eyes and annotations. The results of the comparison are shown in Fig. 4b. Regarding fluorescence intensity, for most images the methodology provided similar results to those obtained from the ground truth from both annotators. Only in eye 5 a systematic difference was observed, in which case the method resulted in slightly lower fluorescence intensity values. More importantly, the change in fluorescence intensity between time points obtained by the method is similar to those of the ground truths. For instance, the Pearson correlation coefficient between the values obtained by the method and those obtained when using ground truth 1 and 2 was, respectively, 0.98 and 0.99. Regarding leakage regions, the results in Fig. 4b show that the detected leakages had similar areas to one of the ground truths for eyes 1 and 2 and slightly larger areas than both ground truths for eye 5. The Pearson correlation coefficient between the areas calculated by the methodology and the areas obtained from ground truth 1 and 2 was, respectively, 0.97 and 0.91 (the average is 0.94).

To quantify the average difference between the results of the leakage identification method and the values obtained using the annotations and verify how this difference compares to inter-annotator changes, the relative difference between each obtained value and one of the ground truths was calculated. Let $L$, $L_{g1}$ and $L_{g2}$ be the leakage fluorescence intensity obtained from, respectively, the leakage identification method, ground truth 1 and ground truth 2. When using ground truth 1 as reference, we calculated the quantities $\left| {L - L_{g1} } \right|/L_{g1}$ and $\left| {L_{g2} - L_{g1} } \right|/L_{g1}$. The former compared the method with ground truth 1, while the latter compared the result of ground truth 2 with ground truth 1. The same was done using ground truth 2 as a reference, in which case we calculate $\left| {L - L_{g2} } \right|/L_{g2}$ and $\left| {L_{g1} - L_{g2} } \right|/L_{g2}$. Each value was then averaged over the 9 annotated images. Table 1 shows the calculated averages as well as standard deviations. The relative difference between the values obtained from the method and those obtained from manual annotations was at most 10% ± 6%. Also, it is clear that the difference between the method and the reference values was comparable to inter-annotator differences. Thus, the method can provide reliable leakage intensity values.

**Table 1:** Comparison between leakage fluorescence intensity values obtained from the method and from the ground truths. Each row corresponds to a ground truth used as reference, while each column indicates the agreement between the respective fluorescence intensity obtained and the reference values.

	Method	Ground truth 1	Ground truth 2
Ground truth 1 as reference	10% ± 6%	0%	8% ± 2%
Ground truth 2 as reference	9% ± 2%	9% ± 2%	0%

The same analysis was applied for comparing the areas of the leakage regions with the ground truths. The results are shown in Table 2.

**Table 2:** Comparison between the total area of the leakage regions obtained from the method and from the ground truths. Each row corresponds to a ground truth used as reference, while each column indicates the agreement between the respective area obtained and the reference values.

	Method	Ground truth 1	Ground truth 2
Ground truth 1 as reference	13% ± 2%	0%	14% ± 2%
Ground truth 2 as reference	21% ± 15%	17% ± 4%	0%

## Discussion

We designed and developed a method for segmenting retinal blood vessels and leakage in FFA images from *Vldlr* knockout mice, a preclinical mouse model with retina blood leakage. A diagram illustrating the steps of the methodology is shown in Fig. 1. In contrast to previous methods in the literature, out method is capable of quantifying the intensity and area of leakage regions at different time points.

Blood leakage in preclinical models of retinal disorders is commonly encountered in research, but its characterization is challenging as the fluorescein signal intensity in leakage regions is weaker than in blood vessels and changes over time after injection. In addition, leakage regions are amorphous, which restricts the definition of prior shape models. The proposed method aimed at first detecting and removing blood vessels and then locating blood leakage regions. It segmented and separated blood leakage from blood vessels and characterized leakage regions. Considering that the FFA images were taken at different time points at slightly distinct locations, the method also provided the registration of the images into a fixed coordinate frame to allow us to compare the blood leakage among the mice under different treatments. The significance of the work lies in improving the consistency and robustness of FFA image analysis, thus providing the means for the advancement of research utilization of FFA in preclinical mouse model.

From Table 1, it is clear that the difference between the method and the reference values is comparable to inter-annotator differences. Thus, the method can provide reliable leakage intensity values. As seen in Table 2, the relative difference between the areas of the leakages measured by the method and ground truth 1 was much lower compared to the difference to ground truth 2. This is likely due to the different criteria used by the annotators for defining the border of a leakage region, as indicated by the large relative difference between the values obtained using the ground truths. This further motivates the usage of a methodology for providing a precise definition of leakage regions, as presented in this study.

One challenge in analyzing FFA imaging of preclinical models is that leakage regions do not have a precise boundary since its intensity varies smoothly in FFA images. It means that even manual markings may change between specialists. This can be seen on the first time point of eye 2 and all time points of eye 5, in which the results for the annotated regions differ. Therefore, one should expect a range of threshold values leading to similar results, and it is often satisfactory to choose a value within this range.

One limitation of this work is that additional structures present in the image due to specific diseases or imaging artifacts might be considered a leakage region. In such cases, an additional preprocessing step needs to be defined to remove such artifacts. Also, a rigid transformation was used for image registration that may not be sufficient for registering images in some cases. In such scenarios, a non-rigid transformation may be more suitable, though it introduces additional parameters for the transformation.

The impact of this work regarding retinal disease research includes the design of algorithms tailored to FFA for preclinical mouse model, *Vldlr*^*−/−*^ mice . For example, Zhao Y. et al. presented a method using graph cut for finding leakage regions in clinical fluorescein angiography of patients who had malarial retinopathy^19^. Though the human images look similar to the images from animal models, there is uniqueness about animal models. For example, at the scale of animal imaging, the leakage regions can take up a large percentage of the whole images, requiring us to develop segmentation methods that can handle images where the foreground (blood vessels and leakage) and background were of comparable sizes. Also, in animal FFA, it is important to image and track the change of leakage, thus we need to judiciously quantify the leakage regions over multiple time points to derive accurate analysis of the dynamic changes of leakage. The first contribution of our work is the design of a segmentation method for separating blood vessels from leakage regions. The second contribution is the tracking and quantification of multiple leakage regions over time points. This feature is particularly useful in preclinical studies as researchers often need to analyze a large number of FFA images within and between groups for comparison, which makes manual and semi-automatic approaches burdensome. Our results show that the proposed method can generate consistent measurements, as can be seen in Fig. 3, whereby all mice had similar quantification of their retinal leakages over time.

For future developments, from the images generated by the method, one can calculate more specific properties, such as the relationship between leakage intensity and blood vessel length or leakage region and blood vessel diameter. Also, with the fast development of deep learning in biomedical image processing, even with retinal images^29^, it is possible to integrate deep learning with the proposed method to achieve improved performance.

## Conclusions

In this work, we designed and tested an image processing pipeline for modeling and quantifying blood vessels and leakage regions in FFA images from *Vldlr* knockout mice, a preclinical mouse model with retina blood leakage. The method can successfully track changes in leakage regions in FFA imaging. Our results showed that the method can achieve good performance in identifying blood vessels and, separately, segmenting leakage regions for quantification. To our knowledge, a methodology for measuring leakage regions has not been proposed before for the imaging protocol considered in our work, which hinders the quantitative comparison with other studies. The proposed method provides researchers who use preclinical models to study retinal diseases a fast and objective quantitative tool for studying FFA images.
